# Potential for Interspecies Toxicity Estimation in Soil Invertebrates

**DOI:** 10.3390/toxics9100265

**Published:** 2021-10-14

**Authors:** Mace G. Barron, Faith N. Lambert

**Affiliations:** Office of Research & Development, U.S. EPA, Gulf Breeze, FL 32561, USA; lambert.faith@epa.gov

**Keywords:** model prediction accuracy, earthworms, interspecies correlation estimation, toxicity extrapolation

## Abstract

Interspecies correlation estimation (ICE) models are linear regressions that predict toxicity to a species with few data using a known toxicity value in a surrogate species. ICE models are well established for estimating toxicity to fish and aquatic invertebrates but have not been generally developed or applied to soil organisms. To facilitate the development of ICE models for soil invertebrates, a database of single chemical toxicity values was compiled from knowledgebases and reports that included 853 records encompassing 192 chemicals and 12 species. Most toxicity data for single chemicals tested in soil media were for species of earthworms, with only limited data for other species and taxa. ICE models were developed for eleven separate species pairs as least squares log-linear regressions of acute toxicity values of the same chemicals tested in both the surrogate and predicted species of soil organisms. Model uncertainty was assessed using leave one out cross-validation as the fold difference between a predicted and measured toxicity value. ICE models showed high accuracy within order (e.g., earthworm to earthworm), but less prediction accuracy in the two across-taxa models (Arthropoda to Annelida and the inverse). This study provides a proof-of-concept demonstration that ICE models can be developed for soil invertebrates.

## 1. Introduction

Interspecies correlation estimation (ICE) models predict toxicity to a species with limited data using a known toxicity value in a surrogate species [1]. The need to evaluate a rapidly growing number of chemicals and efforts to reduce or replace animal testing have increased the need and use of computational toxicity estimation methods, such as ICE models [2]. Most ICE models have been developed as simple linear regressions of acute toxicity values of the same chemicals tested in both the surrogate and predicted species [3]. An extensive technical basis exists for aquatic ICE models including database curation, model validation, comparison to water quality standards, and use in supplementing the taxa diversity of species sensitivity distributions (e.g., Dyer et al. [4]; Awkerman et al. [5]). Suites of ICE models are currently available for fish and aquatic invertebrates, algae, and terrestrial wildlife on the internet platform Web-ICE (www3.epa.gov/webice/), as well as in the literature. Aquatic ICE models are increasingly being used or considered for ecological risk assessment and regulatory applications [2,6,7].

ICE models for soil invertebrates and plants have not been previously available, despite the ecological significance of soil dwelling organisms, and the increasing knowledge base on the toxicity of chemicals to soil organisms. Common soil invertebrate test species include earthworms (e.g., *Eisenia andrei*) and springtails (Collembola; e.g., *Folsomia candida*) [8]. The availability of standardized test methods for some soil invertebrates, such as earthworms (e.g., OECD [9]), could allow many toxicity studies to be performed in a consistent manner. However, the variety of options for acute testing conditions that are recommended within and across species has resulted in inconsistent testing that makes combining data from multiple studies challenging. For example, acute toxicity tests with earthworms can be performed using a 2-day contact toxicity test or a 7–14 day soil exposure.

The objective of the current study was to assess the feasibility of developing ICE models for soil invertebrates to allow for extrapolation between different species and taxa of soil dwelling organisms. Single chemical toxicity data were compiled from the US EPA and simple least squares log-linear regression ICE models were developed as a proof of concept demonstration that ICE models can be developed for soil invertebrates.

## 2. Materials and Methods

### 2.1. Data Compilation

Acute toxicity data for soil invertebrates were obtained from the ECOTOX knowledgebase (https://cfpub.epa.gov/ecotox/; downloaded May 2021), Standartox (http://standartox.uni-landau.de/; downloaded May 2021) [10], and compilations of screening values [11]. The search parameters used to obtain data from ECOTOX and Standartox are included in the Supplementary Materials (Table S1). The ECOTOX knowledgebase, in particular, is a comprehensive database of toxicity values taken primarily from peer-reviewed literature that is reviewed and systematically updated quarterly. Both lethal concentration 50% (LC50) values and lowest observed effect concentrations (LOECs) for mortality were compiled and analyzed separately. Toxicity values were filtered to include only data from experiments that used standard acute soil toxicity test durations (1 day for *C. elegans*, 14 days for earthworms and springtails), endpoints expressed in or convertible to mg/kg soil, and species that had data for at least 3 chemicals. Due to limited data availability, toxicity values for species at any life stage were used and data were not standardized for soil type or environmental conditions (e.g., pH, moisture). All compounds were analyzed as the chemical form originally reported (e.g., metal salts were not converted to toxicity of the elemental form). The resulting toxicity database included 853 records encompassing 197 chemicals and 12 species (Table 1). Prior to model development, species mean acute values (SMAVs) were calculated as the geometric mean of toxicity values (LC50 or LOEC) for each endpoint, species, and chemical. SMAVs were then used in ICE models.

### 2.2. Model Development

ICE models were developed using least squares log-linear regression, as described by Raimondo et al. [3], by pairing toxicity values by chemical between the surrogate species and predicted species [3]. ICE models describe the relationship of chemical sensitivity between two species by the equation Log10(*Predicted Toxicity*) = *a* × Log10(*Surrogate Toxicity*) + *b* [3]. For the soil invertebrate ICE models, *Predicted toxicity* was the SMAV of the species with unknown chemical toxicity, *Surrogate Toxicity* was the SMAV of the surrogate species, *a* was the slope of the regression line, and *b* was the intercept of the regression.

Models were developed separately for SMAVs derived from LOEC and LC50 data. A separate model was developed for each potential pair of species that had data for the same endpoint for at least three common chemicals. The *R^2^* and mean square error (MSE) of each model were calculated as a measure of model fit and model error, respectively. All models were developed using the stats package in R.

### 2.3. Model Prediction Accuracy

Significant models (*p* < 0.05) that had at least four data points were evaluated using leave-one-out cross validation (LOOCV), which has previously been employed for evaluating model accuracy [3,12]. In LOOCV, one data point is removed from the dataset. Then, a new model is built with the remaining values and used to predict the removed value. This was systematically reiterated for each *Predicted toxicity* value in each model. LOOCV analysis was conducted using the caret package in R [13]. The fold differences of removed values were calculated by dividing the non-transformed estimated value by the actual value or vice versa, if the latter was larger. The percentage of estimates that fell within 5-fold of the actual value was calculated for each model and used as a prediction success rate. As mentioned in Willming et al. [12], values within 5-fold of each other are within the range of interlaboratory variation.

## 3. Results

### 3.1. Within-Taxa Models

Available data allowed for the development of within-taxa models between earthworm species (same Order: Opisthopora). Using least-squares log-linear regression, a total of 3 significant (*p* < 0.05) model pairs were developed using LC50 data (out of 7 pairs total) (Table 2) and 6 significant (*p* < 0.05) models were developed using LOEC data (out of 14 total) (Table 3). LOOCV analysis determined that within-species models predicted removed values within 5-fold of the actual toxicity value 99% of the time and 97% of the time when using LC50 values and LOEC values, respectively.

### 3.2. Across-Taxa Models

Only two across-taxa models (different Phylums) were significant (*p* < 0.05). Models were between *Folsomia candida* (Phylum: Arthropoda, Class: Collemobola) and *Eisenia fetida* (Phylum: Annelida, Class: Clitellata) (Table 4). LOOCV analysis determined that across-taxa models predicted removed LOEC values within 5-fold of the actual toxicity value 62.5% of the time, on average.

## 4. Discussion

The current study was a proof-of-concept demonstration that ICE models can be developed for soil invertebrates. ICE models showed high prediction accuracy within the Order Opisthopora (i.e., earthworm to earthworm), but lower accuracy in the two across-taxa models (Arthropoda to Annelida and the inverse). These initial results are similar to observations of ICE model extrapolation in aquatic species, where increasing taxonomic distance results in less prediction accuracy and generally greater uncertainty [12,14]. The soil invertebrate ICE models were developed with compounds from multiple chemical classes, which can reduce model accuracy for pairs of species with limited taxa relatedness. Research with aquatic toxicity ICE models has shown that across-taxa predictions can be improved by developing models using only compounds with the same mode of action (MOA) and same structural class [3]. Additional data would be needed to develop a suite of single chemical class-specific models and to more comprehensively explore whether these models could improve cross-taxa extrapolation in soil invertebrates, but were not available in the explored databases.

Soil invertebrate toxicity data used in this study were not curated beyond acute toxicity endpoint and were not standardized for test conditions, which can result in greater uncertainty in ICE models and lower prediction accuracy [15]. Although toxicity values from knowledgebases such as ECOTOX are typically used without further curation in environmental toxicology applications, it can lead to greater uncertainty and less prediction accuracy in model development [10,15,16]. For example, Hrovat et al. [17] reported orders of magnitude variability in fish acute toxicity values and incomplete records in nearly 70% of the 4654 ECOTOX records assessed. Toxicity data in the current study were not standardized for soil type, which can substantially influence species sensitivity of soil organisms, particularly for ionizable chemicals, such as metals, and phenolics affected by pH and clay content [18]. However, the availability of standardized test methods should provide for datasets of more consistently collected toxicity data (e.g., OECD [9]). Future research should determine if standardization for soil chemistry and physical properties would reduce uncertainty in interspecies extrapolation modeling, particularly for species pairs with large taxonomic distance.

One surprising aspect of this study was the generally limited toxicity data for soil invertebrates from single chemicals tests in soil media. Additional data for single chemical tests are available as short-term contact toxicity tests, but these were excluded from the current study because of apparent lack of ecological relevance to soil exposures. Bioassays of field collected samples are more routinely collected as part of contaminated site assessments (e.g., Reinecke et al. [19]; Fründ et al. [20]), but single chemical test data appear to be rarely reported based on current knowledgebase searches. Earthworm tests dominate the soil toxicity data from the comprehensive ecotoxicity databases used for source data, with few data for other taxa. Given their ecological importance and prevalence of soil contamination globally, the availability of additional toxicity data for soil invertebrates is critically needed and would allow for the expanded development of ICE models and application in ecological assessments. In particular, toxicity data for a wider diversity of species may benefit the development of benchmark values, such as ecological soil screening levels (EcoSSL), by better representing soil ecosystem biodiversity.

## Figures and Tables

**Table 1:** Summary of compiled soil invertebrate toxicity values from single chemicals tested in soil medium.

Endpoint	Common Name	Taxa	Species	Number of Chemicals
LC50	Earthworm	Annelid	*Eisenia andrei*	35
	Earthworm	Annelid	*Eisenia fetida*	162
	Earthworm	Annelid	*Lumbricus rubellus*	15
	Earthworm	Annelid	*Lumbricus terrestris*	26
	Nematode	Nematode	*Caenorhabditis elegans*	10
	Pot worm	Annelid	*Enchytraeus albidus*	3
	Springtail	Arthropod	*Heteromurus nitidus*	5
	Woodlouse	Arthropod	*Porcellionides pruinosus*	3
LOEC	Earthworm	Annelid	*Allolobophora tuberculata*	9
	Earthworm	Annelid	*Eisenia andrei*	24
	Earthworm	Annelid	*Eisenia fetida*	26
	African Earthworm	Annelid	*Eudrilus eugeniae*	9
	Earthworm	Annelid	*Lumbricus rubellus*	14
	India Blue Earthworm	Annelid	*Perionyx excavatus*	9
	Springtail	Arthropod	*Folsomia candida*	9
	Springtail	Arthropod	*Heteromurus nitidus*	4

**Table 2:** Significant within-taxa models (same Order: Opisthopora) using single chemical median lethal concentration (LC50) data for soil invertebrates. Model parameters included intercept, slope, and *R*^2^, the model *p*-value, mean square error (MSE), and the total number of data points (*n*). The cross-validation within 5-fold describes the prediction success rate, or percentage of estimated toxicity values from leave one out cross validation that were within 5-fold of the measured LC50 value. Numbers in the left-most column indicate model pairs which use the same two species, alternating which is used as the surrogate.

Model Pair	Predicted Species (*y*-axis)	Surrogate Species (*x*-axis)	Intercept	Slope	*R* ^2^	*p*-Value	MSE	*n*	Cross- Validation within 5-Fold
1	*Eisenia andrei*	*Eisenia fetida*	0.37	0.72	0.63	4.50 × 10^−5^	0.20	19	100%
	*Eisenia fetida*	*Eisenia andrei*	0.34	0.88	0.63	4.50 × 10^−5^	0.24	19	100%
2	*Eisenia fetida*	*Lumbricus terrestris*	0.97	0.60	0.36	3.07 × 10^−3^	0.28	22	100%
	*Lumbricus terrestris*	*Eisenia fetida*	0.96	0.60	0.36	3.07 × 10^−3^	0.28	22	100%
3	*Eisenia andrei*	*Lumbricus terrestris*	0.34	0.72	0.39	3.03 × 10^−2^	0.42	12	100%
	*Lumbricus terrestris*	*Eisenia andrei*	1.09	0.54	0.39	3.03 × 10^−2^	0.32	12	91.67%

**Table 3:** Significant within-taxa models (same Order: Opisthopora) using single chemical lowest observed effect concentration (LOEC) data. Model parameters included intercept, slope, and *R*^2^, the model *p*-value, mean square error (MSE), and the total number of data points (*n*). The cross-validation within 5-fold describes the prediction success rate, or percentage of estimated toxicity values from leave one out cross validation that were within 5-fold of the measured LOEC value. Numbers in the left-most column indicate model pairs which use the same two species, alternating which is used as the surrogate.

Model Pair	Predicted Species (*y*-axis)	Surrogate Species (*x*-axis)	Intercept	Slope	*R^2^*	*p*-Value	MSE	*n*	Cross- Validation within 5-Fold
1	*Eudrilus euganiae*	*Eisenia fetida*	−0.07	1.09	0.60	8.77 × 10^−3^	0.26	10	90%
	*Eisenia fetida*	*Eudrilus euganiae*	1.00	0.55	0.60	8.77 × 10^−3^	0.13	10	100%
2	*Eudrilus euganiae*	*Allolobophora tuberculata*	–0.27	1.04	0.96	4.48 × 10^−6^	0.02	9	100%
	*Allolobophora tuberculata*	*Eudrilus euganiae*	0.35	0.92	0.96	4.48 × 10^−6^	0.02	9	100%
3	*Perionyx excavatus*	*Eisenia fetida*	0.27	0.94	0.58	1.03 × 10^−2^	0.21	10	90%
	*Eisenia fetida*	*Perionyx excavatus*	0.83	0.62	0.58	1.03 × 10^−2^	0.14	10	100%
4	*Eisenia fetida*	*Allolobophora tuberculata*	0.59	0.67	0.74	1.50 × 10^−3^	0.09	10	90%
	*Allolobophora tuberculata*	*Eisenia fetida*	0.06	1.09	0.74	1.50 × 10^−3^	0.14	10	90%
5	*Perionyx excavatus*	*Allolobophora tuberculata*	0.09	0.90	0.95	7.88 × 10^−6^	0.02	9	100%
	*Allolobophora tuberculata*	*Perionyx excavatus*	0.03	1.05	0.95	7.88 × 10^−6^	0.02	9	100%
6	*Eudrilus euganiae*	*Perionyx excavatus*	–0.31	1.13	0.96	2.84 × 10^−6^	0.02	9	100%
	*Perionyx excavatus*	*Eudrilus euganiae*	0.36	0.86	0.96	2.84 × 10^−6^	0.01	9	100%

**Table 4:** Significant across-taxa models (different Phylum) using lowest observed effect concentration (LOEC) data. Model parameters included intercept, slope, and *R*^2^, model *p*-value, mean square error (MSE), and the total number of data points (*n*). The cross-validation within 5-fold describes the prediction success rate, or percentage of estimated toxicity values from leave one out cross validation that were within 5-fold of the measured LOEC value. Numbers in the left-most column indicate model pairs which use the same two species, alternating which is used as the surrogate.

Model Pair	Predicted Species (*y*-axis)	Surrogate Species (*x*-axis)	Intercept	Slope	*R* ^2^	*p*-Value	MSE	*n*	Cross-Validation within 5-Fold
1	*Folsomia candida*	*Eisenia fetida*	0.85	0.47	0.93	3.37 × 10^−2^	0.04	4	50%
	*Eisenia fetida*	*Folsomia candida*	–1.55	2.00	0.93	3.37 × 10^−2^	0.15	4	75%

## Data Availability

Data supporting reported results is included in the Supplementary Materials.

## Supplementary Materials

The following are available online at https://www.mdpi.com/article/10.3390/toxics9100265/s1, Table S1: Summary of search parameters used in the ECOTOX knowledgebase and Standartox databases to obtain toxicity values for use in this study. Spreadsheet S1: Soil ICE database which includes all toxicity data used in this study.
