# Supplementary material for: Potential for Interspecies Toxicity Estimation in Soil Invertebrates
Source: Toxics. 2021 Oct 14;9(10):265. doi: 10.3390/toxics9100265 (PMC8541476; doi:10.3390/toxics9100265)
Supplement: Supplementary file 1 [file toxics-09-00265-s001.zip › table S1.pdf]

Supplementary Materials: Potential for Interspecies Toxicity Estimation in Soil Invertebrates

Mace G. Barron and Faith N. Lambert

Table S1. Summary of search parameters used in the ECOTOX knowledgebase and Standartox databases to obtain toxicity values for use in this study. Data was further curated after collection as described in the text.

Database	Date Accessed	Filter Category	Parameter Group	Name	Value
ECOTOX knowledgebase (for LC50 data) (https://cfpub.epa.gov/ecotox/)	6/4/2021	Habitat	Terrestrial	Terrestrial	Terrestrial
		Effect Measurements	Mortality Group	Mortality	ALL
		Endpoints	Concentration Based Endpoints	LD50	ALL
				LD50	ALL
				LC/LD xx (all% values)	ALL
		Species	Animals	Insects/Spiders	ALL
				Other Invertebrates	ALL
				Crustaceans	ALL
				Molluscs	ALL
				Worms	ALL
		Test Conditions	Exposure Media	Soil - Unspecified Soil	ALL
				Soil - Natural Soil	ALL
				Soil - Litter	ALL
				Soil - Manure	ALL
				Soil - Mineral Soil	ALL
				Soil - Soil Mixture	ALL
				Soil - Artificial	ALL
				Soil - Humus	ALL
			Exposure Types	Both Aquatic and Terrestrial - Environmental	ALL
			Test Locations	Lab	ALL

Table S1. *Continued.*

Database	Date Accessed	Filter Category	Parameter Group	Name	Value
ECOTOX knowledgebase (for LOEC data) (https://cfpub.epa.gov/ecotox/)	6/23/2021	Habitat	Terrestrial	Terrestrial	Terrestrial
		Effect Measurements	Mortality Group	Mortality	ALL
		Endpoints	Concentration Based Endpoints	LOEC	ALL
				LOEL	ALL
		Species	Animals	Insects/Spiders	ALL
				Other Invertebrates	ALL
				Crustaceans	ALL
				Molluscs	ALL
				Worms	ALL
		Test Conditions	Exposure Media	Soil - Unspecified Soil	ALL
				Soil - Natural Soil	ALL
				Soil - Litter	ALL
				Soil - Manure	ALL
				Soil - Mineral Soil	ALL
				Soil - Soil Mixture	ALL
				Soil - Artificial	ALL
				Soil - Humus	ALL
			Exposure Types	Both Aquatic and Terrestrial - Environmental	ALL

			Test Locations	Lab	ALL
Standartox (http://standartox.uni-landau.de/)	6/4/2021	Chemicals	Concentration Unit	mg/kg	checked
		Taxa	Habitat	Terrestrial	checked
			Trophic Level	Heterotroph	checked
			Ecotoxicological	Invertebrate	checked
		Test	Durations from		NA
			to(h)		NA
			Exposure group	Environmental	checked
			Endpoint	XX50	checked
			Effect group	Mortality	checked
